# Genome-wide imputation using the practical haplotype graph in the heterozygous crop cassava

**DOI:** 10.1093/g3journal/jkab383

**Published:** 2021-11-09

**Authors:** Evan M Long, Peter J Bradbury, M Cinta Romay, Edward S Buckler, Kelly R Robbins

**Affiliations:** 1 Plant Breeding and Genetics Section, School of Integrative Plant Science, Cornell University, Ithaca, NY 14853, USA; 2 Institute for Genomic Diversity, Cornell University, Ithaca, NY 14853, USA; 3 United States Department of Agriculture-Agricultural Research Service, Robert W. Holley, Center for Agriculture and Health, Ithaca, NY 14853, USA

**Keywords:** cassava, imputation, haplotype, practical haplotype graph, genomic prediction, heterozygous, Beagle

## Abstract

Genomic applications such as genomic selection and genome-wide association have become increasingly common since the advent of genome sequencing. The cost of sequencing has decreased in the past two decades; however, genotyping costs are still prohibitive to gathering large datasets for these genomic applications, especially in nonmodel species where resources are less abundant. Genotype imputation makes it possible to infer whole-genome information from limited input data, making large sampling for genomic applications more feasible. Imputation becomes increasingly difficult in heterozygous species where haplotypes must be phased. The practical haplotype graph (PHG) is a recently developed tool that can accurately impute genotypes, using a reference panel of haplotypes. We showcase the ability of the PHG to impute genomic information in the highly heterozygous crop cassava (*Manihot esculenta*). Accurately phased haplotypes were sampled from runs of homozygosity across a diverse panel of individuals to populate PHG, which proved more accurate than relying on computational phasing methods. The PHG achieved high imputation accuracy, using sparse skim-sequencing input, which translated to substantial genomic prediction accuracy in cross-validation testing. The PHG showed improved imputation accuracy, compared to a standard imputation tool Beagle, especially in predicting rare alleles.

## Introduction

The past decade has seen an abundance of genomic sequence data produced for research and application in agricultural crops. With these new technologies, comes a question on how to effectively implement them (Torkamaneh *et al.* 2018). Two of the most common uses of genome-wide sequence data are genomic selection (GS) and genome-wide association studies (GWAS). While most GWAS attempt to locate distinct, causative regions of the genome, GS incorporates all available markers to predict plant traits (Meuwissen *et al.* 2001). GS leverages a training set population that has both genotypic and phenotypic data to predict traits in a related germplasm with only genotypic data (Heffner *et al.* 2009). This allows breeders to both increase accuracy in selecting traits with low heritability and accelerate the rate of selections by decreasing selection cycle time (Xu *et al.* 2020). 

While sequencing data have become increasingly common in agricultural applications, the financial cost remains a challenge to widespread implementation. Reduced representation marker systems have been produced to limit costs of performing genomic analyses (Romay 2018), all of which vary in marker density and depth, cost, and genotype confidence. In scenarios with limited diversity, such as single breeding pools or postbottleneck populations, individuals share large stretches of sequence. The strong association between alleles in these blocks, or their linkage disequilibrium (LD), determines the number and distribution of genotype markers needed to explain the genetic variation in the population. High density of markers becomes more important when performing analyses in populations where LD decays quickly as in species with high diversity or among unrelated individuals. High marker density can also be beneficial to incorporate knowledge on previously studied loci across the genome.

To affordably obtain high-density genotypes or to bridge information between different marker platforms it becomes necessary to impute missing genotypes from available genotype data. Increasing the stability across genotyping platforms and reducing per-sample costs, becomes even more relevant in plant breeding scenarios, where many thousands of offspring are evaluated and changes in marker platform are common. Computational techniques to impute genome-wide information have been produced to bridge genotypic information from different marker panels and augment genotypic information from limited inputs (Yun *et al.* 2009). Genomic imputation methods often rely on a related training set with high confidence genotypic information to then predict missing genotypes. These methods have been shown to improve consistency and efficiency of analyses of both genome-wide associations (Spencer *et al.* 2009) and GS (Cleveland *et al.* 2011).

Imputation is very common in genomic studies but is still plagued with barriers to high accuracy in many species. Known limitations of imputation stem from LD, allele frequencies, and population structure of the training population (Alipour *et al.* 2019). These difficulties are further compounded when working with a highly heterozygous crop, where both copies of the genome need to be modeled (Fragoso *et al.* 2016; Nazzicari *et al.* 2016). Heterozygosity introduces the challenge of phasing, the process assigning alleles to haplotypes, a challenge that is not limited to plants (Friedenberg and Meurs 2016). Imputation accuracy has been shown to affect the accuracy of genomic prediction in multiple scenarios (Pimentel *et al.* 2015; Wang *et al.* 2016; Van Den Berg *et al.* 2017). In addition, when tracking causative variation through the genome, high accuracy in imputation is necessary to evaluate variation across the entire genome. Highly accurate imputation methods are needed to increase the gains made by GS by making genotyping cheaper, more accurate, and more consistent.

It has been shown that rare variants contribute to the genetic load and overall performance of crops (Yang *et al.* 2017; Kremling *et al.* 2018; Kono *et al.* 2019), making high imputation accuracy, especially for alleles at low frequency, desirable for plant genomics applications. Diverse imputation tools exist and are often designed for different scenarios. One of the more common tools Beagle (Browning *et al.* 2018), which was designed for application in humans, works by leveraging LD between variants to predict missing genotypes. Beagle uses LD clustering to create an acyclic graph and a Hidden Markov model (HMM) to infer the most likely haplotype. Another method EAGLE leverages stretches of identity by descent (IBD) to perform long-range phasing (Loh *et al.* 2016). In humans, where these imputation algorithms have been showcased, they have the advantage of large datasets with data from several thousands of individuals (Loh *et al.* 2016; Browning *et al.* 2018), while this is not often possible in many plant breeding scenarios.

In maize, it’s been shown that Beagle has difficulty accurately imputing rare variants, while a haplotype library-based method such as FILLIN can do so more easily (Swarts *et al.* 2014). A recently developed method known as the practical haplotype graph (PHG) was created to leverage known haplotypes in a graph structure to efficiently impute genotypes. The PHG simplifies the genome to a set of distinct regions of the genome, for which it defines haplotypes. These haplotypes are constructed from whole-genome sequence data or genome assemblies and are used to construct a trellis graph, capturing the diversity of haplotypes at each range and the relationships between adjacent haplotype regions. Sequence reads are then aligned to the graph and an HMM is applied to predict the most likely haplotypes. By aligning reads to pan-genome haplotypes, the PHG minimizes errors due to reference bias, poor alignment, and miscalled variants. Utilizing a PHG methodology in plant and animal applications can improve the quality and quantity of genotype data for use in breeding and mapping scenarios.

Here, we showcase the potential application of the PHG in imputation of heterozygous crops. The PHG has already been shown to be an efficient tool for aiding imputation and GS in breeding of the inbred cereal crop Sorghum (Jensen *et al.* 2020). It has also been implemented to impute genotypes in highly diverse maize lines (Franco *et al*. 2020). To show the utility of the PHG in a heterozygous crop we must overcome two distinct challenges: obtaining phased haplotypes to populate the database and modeling both copies of the genome accurately. Without an abundance of data, it is very difficult to obtain accurate phasing in a highly heterozygous species. This study will explore these challenges by imputing haplotypes from low-coverage skim sequencing, while comparing results to Beagle’s performance.

To investigate the construction and performance of the PHG in a heterozygous scenario, we created a PHG for cassava (*Manihot esculenta*), a root crop with high levels of heterozygosity reinforced by centuries of clonal propagation. In this study, we utilize sequence data from the previously published HapMapII in cassava (Ramu *et al.* 2017), which includes WGS data for 241 cassava clones. These data are used to produce a PHG in cassava and showcase its effectiveness in genomic imputation in a heterozygous crop. We further validate these methods through genomic prediction and simulation.

## Materials and methods

### Haplotype sampling

Genomic data were used from the second-generation Cassava Haplotype map consisting of 241 taxa, including both cultivated and wild germplasm (Ramu *et al.* 2017). Raw data are composed of short-read, whole-genome sequence data from each taxon amounting to greater than 20× coverage on average. The high depth of the sequence data is necessary to accurately distinguish between heterozygous and homozygous variants. We used the cassava v6 reference genome assembly in this study, which contains 18 chromosome level scaffolds summing to ∼500 Mbp of the estimated genome size of 700 Mbp. Haplotype regions, termed here as reference ranges, were defined by genic regions with additional 1000 bp flanking sequence resulting in ∼32,000 reference ranges after merging overlapping ranges, with an average size of 4 kbp.

**Figure 1:**
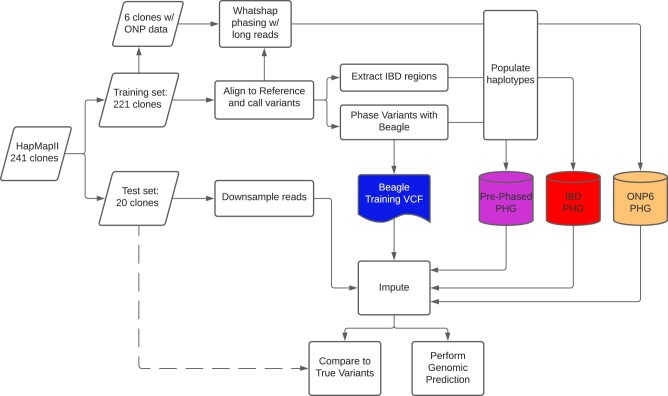
Imputation Methodology Flowchart. Diagram of methods used for the building PHG databases and performing imputation evaluations.

**Figure 2:**
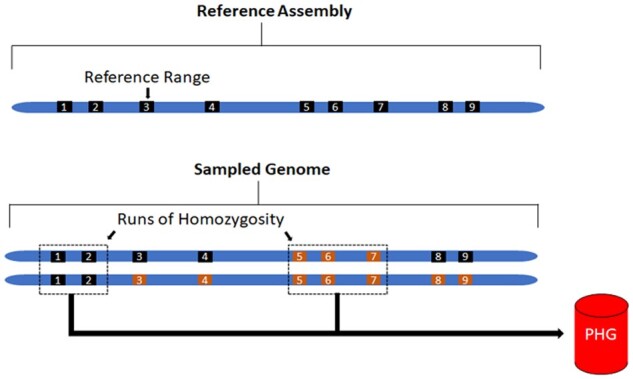
Haplotype view of the genome. (Top) Representation of reference ranges informed from genic regions from the reference genome. (Bottom) haplotypes sampled from runs of homozygosity for use in PHG with different colors representing separate haplotypes at a given region (*i.e.*, ranges 1, 2, 5, 6, 7 are homozygous and haplotypes can be sampled).

The detailed process of creating a PHG is outlined at “https://bitbucket.org/bucklerlab/practicalhaplotypegraph/wiki/Home” and has been described previously (Jensen *et al.* 2020; Valdes Franco 2020). Here, we outline the specific steps taken to create a PHG in the heterozygous crop cassava (Figure  1). The major hurdle to producing a haplotype graph in a heterozygous species is obtaining accurately phased haplotypes. Because many of these cassava lines are cultivated taxa, we expected to find IBD haplotypes brought about by generations of breeding within restricted breeding pools. These IBD segments provide confidently phased haplotypes as well as capturing their relationships to adjacent haplotypes (Figure  2). We identified and sampled these homozygous haplotypes which we inferred to represent IBD haplotypes. This was done by measuring the number of heterozygous variants for each reference range in each taxon, then classifying those haplotypes as homozygous or not. The threshold for haplotypes to be considered IBD was determined empirically to be 0.001 heterozygous SNPs per base pair (Supplementary Figure S1), as *de novo* mutations or errors in variant calling may produce low levels of perceived heterozygosity. This threshold was additionally validated by testing imputation accuracy of the PHG.

After haplotypes were sampled from IBD regions of the genome, they were loaded as GVCF files into a PHG database. Similar haplotypes were then collapsed based on sequence similarity to produce a representative set of available haplotypes. Haplotypes are collapsed to make alignment more efficient, while retaining as much distinct haplotype information as possible. Collapsing is performed using an unweighted pair group method with arithmetic mean (upgma) tree from pairwise distance matrix from sequence variants to measure the similarity between haplotypes. Based on imputation accuracy tests, we chose a level of similarity (PHG parameter: maximum divergence) to collapse haplotypes of 0.001, corresponding to less than 1 in 1000 nucleotide differences between haplotypes. This level of collapsing maintains high accuracy while collapsing redundant haplotypes (Supplementary Figure S2). We then produced a pan-genome composed of consensus haplotypes representing the diversity of haplotypes.

### Predicting haplotypes

Once we obtained a set of consensus haplotypes, we implemented an HMM to infer genome-wide haplotypes from low-depth genotyping data. Sparse genotype information was created by downsampling whole-genome sequence data randomly using samtools to simulate skim sequencing. We randomly sampled 20 taxa from the cultivated varieties within the population to serve as a test set for downstream analyses, while using the remaining 221 clones for haplotype sampling. To test different levels of sequencing depth, we downsampled reads to amounts estimated to represent 0.1×, 0.5×, 1×, 5×, and 10× single-end, whole-genome sequence coverage. In addition, we tested imputation using available Genotype-By-Sequencing (GBS) data for these lines.

These sampled sequences were aligned to the consensus haplotypes stored in the PHG to impute whole-genome variants. A trellis graph is formed with every reference range representing separate ranges and the consensus haplotypes as nodes at each of those ranges. The most likely paths through the graph were then determined using an HMM Viterbi algorithm. Because cassava is heterozygous and diploid, this step produces the two most likely paths for each taxon. The emission and transition probability parameters of the HMM are defined by the genomes of the reference population used to build the database. The emission probabilities are calculated by considering the probability of two given haplotypes, given the aligned reads. The transition probabilities are defined by the edges between haplotypes in the PHG.

Due to the sparse sampling of IBD haplotypes from heterozygous taxa used to produce the PHG, the database lacked abundant transition information between adjacent reference ranges. To compensate for this, we aligned WGS for all 241 taxa used to create the database and predicted most likely paths through the graph. These paths were then used to augment the transition probabilities, without contributing any additional haplotypes.

### Beagle imputation

We compared our imputation accuracy results to the common genotype imputation tool Beagle (Browning *et al.* 2018). Beagle was developed for the purpose of human data, but is a common tool used by many plant studies to impute missing genotypes. Because Beagle v4 has the ability to incorporate genotype likelihoods based on read depth, we used it for the imputation of the low-depth sequence when it improved accuracy, otherwise we utilized Beagle v5. We used the same HapMapII data from the 241 clones to impute missing genotypes with Beagle.

### Genomic prediction

We used 57 clones from a single breeding program, to reduce effects of population structure, to determine the impact of imputation errors on genomic prediction accuracy using cross-validation. Reads were downsampled and imputed as previously described. Three root traits were used for genomic cross-validation: fresh root yield, root size, and root number. Phenotypes for each clone were downloaded from CassavaBase.org, constituting 57 clones, spanning 23 years from 1996 to 2018, across 13 locations in Africa. Tenfold cross-validation was performed by randomly selecting 10% of the clones to hold out and predict using the remaining clones as a training set. The correlation between predicted phenotype and the observed best linear unbiased estimate was used as the prediction accuracy. We performed 50 replications as well as a single holdout prediction to measure genomic prediction accuracy. A single-step model was performed:

$$\begin{array}{c} \hat{y}=\mu +G_{i}+B_{j}+R_{k}+L_{l}+Y_{m}+G_{i}XL_{l}+G_{i}XY_{m}\\ G_{i}∼\;N(0,G\sigma_{G}^{2}),\;B_{j}∼N(0,I\sigma_{B}^{2}),R_{k}∼N(0,I\sigma_{R}^{2}),L_{l}∼N(0,I\sigma_{l}^{2}),\\ Y_{m}∼N(0,I\sigma_{m}^{2}) \end{array}.$$

Here, $\hat{y}$ is the predicted trait and ***μ*** is the fixed effect of the overall mean. Random effects were fitted as follows: ***G*** is genotype effect of the *i*th clone, ***B*** is the effect of the *j*th block, **R** is the effect of the *k*th replicate, **L** is the location of the *l*th location, ***Y*** is the effect of the *m*th year, ***GXL*** is the interactive effect of the *i*th clone and the *l*th location, and ***GXY*** is the interaction effect of the *i*th clone and the mth year. This was performed using the mixed model tool Echidna (Gilmour 2019).

### Prephased haplotype PHG

We investigated the viability of using computationally phased haplotypes to curate a PHG database rather than relying on IBD regions of the genome. First, we phased the variants from the 241 cassava clones using a combination of Beagle (Browning *et al.* 2018) and HAPCUT2 (Edge *et al.* 2017). These variants were used to create a PHG to be tested against the IBD version of the PHG. The second test utilized Oxford Nanopore (ONP) long-read sequencing from six cassava clones within the HMII population. High molecular weight DNA was extracted from young cassava leaves, selected for fragments 20–80 kbp long, and sequenced with MinION following the manufacturer recommendations. Variants were called using Guppy and their variants phased with WhatsHap (Schrinner *et al.* 2020a). These six clones were then used to populate another PHG, we will identify as the “ONP6 PHG”. Larger reference ranges were divided into smaller regions to increase the probability of sampling correctly phased haplotypes. Twenty clones with the highest relationship to the six taxa with ONP data were used as the test set for these tests.

### Imputation from simulated genotypes

**Figure 3:**
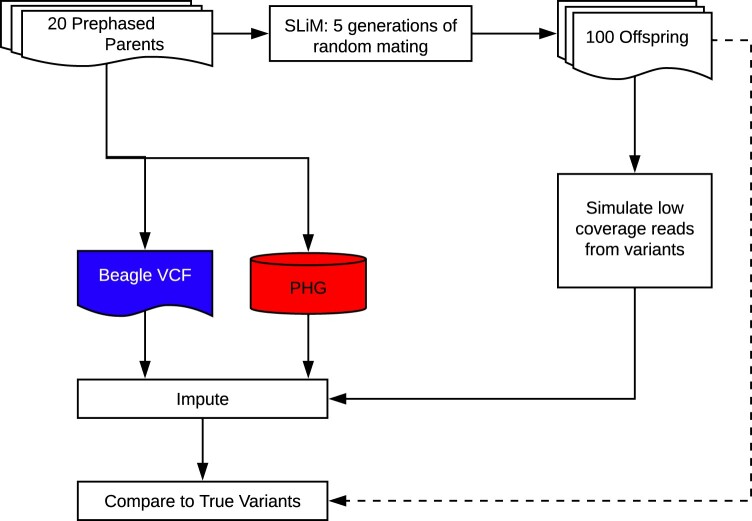
Simulation methodology flowchart. Diagram of simulation scheme showing how simulated offspring were generated and used to test imputation accuracy under ideal haplotype sampling scenarios.

A sample of 20 related individuals from the HapMapII population was selected to serve as parents for a simulated genotyping scenario. The genomes were phased using Beagle and then used to populate a PHG database. We then used these parents to simulate five generations of random mating given a population size of 100 (Figure  3). Forward genetic simulations were completed using SLiM (Haller and Messer 2019). Artificial short-read sequencing was then simulated for these offspring using neat-genreads (Stephens *et al.* 2016) at varied coverage levels. Reads were then aligned using bwa used to call and impute variants using Sentieon (Kendig *et al.* 2019) and Beagle. Reads were also aligned to the PHG formed from the original parents for imputation.

## Results

### Haplotype sampling

To obtain phased haplotypes for the PHG, we sampled haplotypes from homozygous regions of each clone. Centuries of clonal propagation and reported inbreeding depression (de Freitas *et al.* 2016) suggest cassava germplasm would be highly heterozygous, however, we found that, on average, ∼20% of all reference ranges from each taxon were homozygous. This resulted in a high number of missing haplotypes in each taxon, but a high confidence in the phased haplotypes that were sampled. Despite the variability in the number of homozygous samples by reference range, >90% of the reference ranges were homozygous in at least 10% of the HapMapII population (Supplementary Figure S3). From these IBD haplotypes, we were able to sample ∼50% of the segregating sites. This proportion increased to 77% when considering sites with minor allele frequency above 5%, suggesting that many of the common variable sites have been sampled.

### Imputation and genomic prediction accuracy

Because imputation accuracy is dependent on the relative allele frequency and phase of the allele being called, we classified genotype calls by allele frequency class: homozygous major (both alleles are identical and have >50% allele frequency in HapMapII), homozygous minor (both alleles are identical and have <50% allele frequency in HapMapII), and heterozygous (two different alleles are present). In our analyses, imputation accuracy is defined as the ability of the imputation method to reconstitute genome-wide SNPs from the input data. We use the correlation between the predicted alleles and the true alleles (defined by HapMapII) as a metric to make the PHG and Beagle comparable, because the PHG utilizes reads and Beagle utilizes variants to make their predictions.

**Figure 4:**
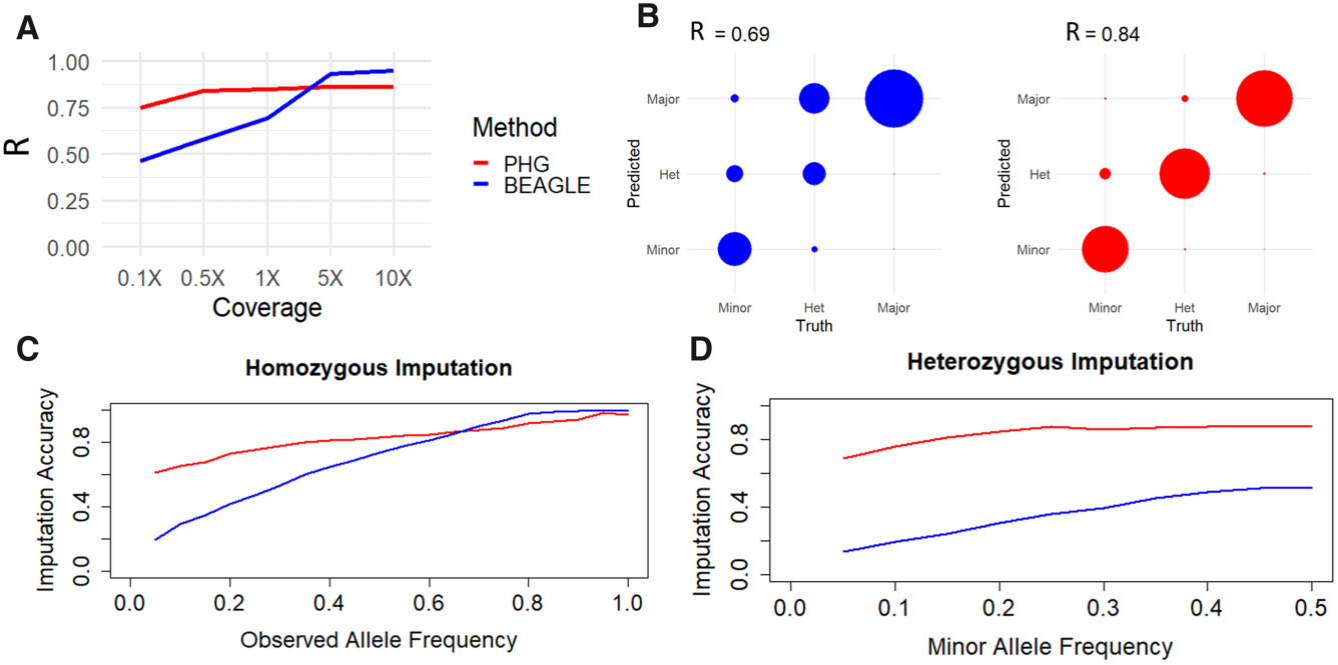
Imputation accuracy from skim sequencing. (A) Displays correlation between imputed and true variants by imputing with the PHG and Beagle at different levels of skim sequencing. (B) Displays concordance between true and imputed alleles at 1× coverage separated by alleles classes: minor, heterozygous, and major (circle radius is equal to the proportion of alleles in each class). (C) Imputation accuracy at 1× coverage is shown for homozygous genotypes separated by allele frequency of the true allele at that locus. (D) Imputation accuracy at 1× coverage is shown for heterozygous genotypes separated by minor allele frequency at that locus.

Imputation of skim sequence genotyping showed PHG methods had a large advantage over Beagle using low-coverage sequence. At a level of 1× coverage random sequencing, the PHG predicted allele calls with a correlation of *R*^2^ = 0.84, while the correlation between Beagle predicted alleles and the true calls was *R*^2^ = 0.69 (Figure  4A). At higher depths of coverage (>5×), the raw data provide ample information to distinguish between homozygous and heterozygous genotypes, allowing Beagle to determine the correct genotype. The PHG, however, is able to distinguish between the available haplotypes at a coverage of 0.5× and adding additional sequence data does not increase the accuracy, as there is no correlation between accuracy and coverage beyond 0.5×.

The improved performance of the PHG is most noticeable in its accurate predictions of heterozygous and rare genotypes. The PHG was able to impute genotypes with high accuracy regardless of allele class (Figure  4B). The PHG’s high accuracy at low allele frequencies for both homozygous (Figure  4C) and heterozygous genotypes (Figure  4D) display its ability to impute rare alleles.

**Figure 5:**
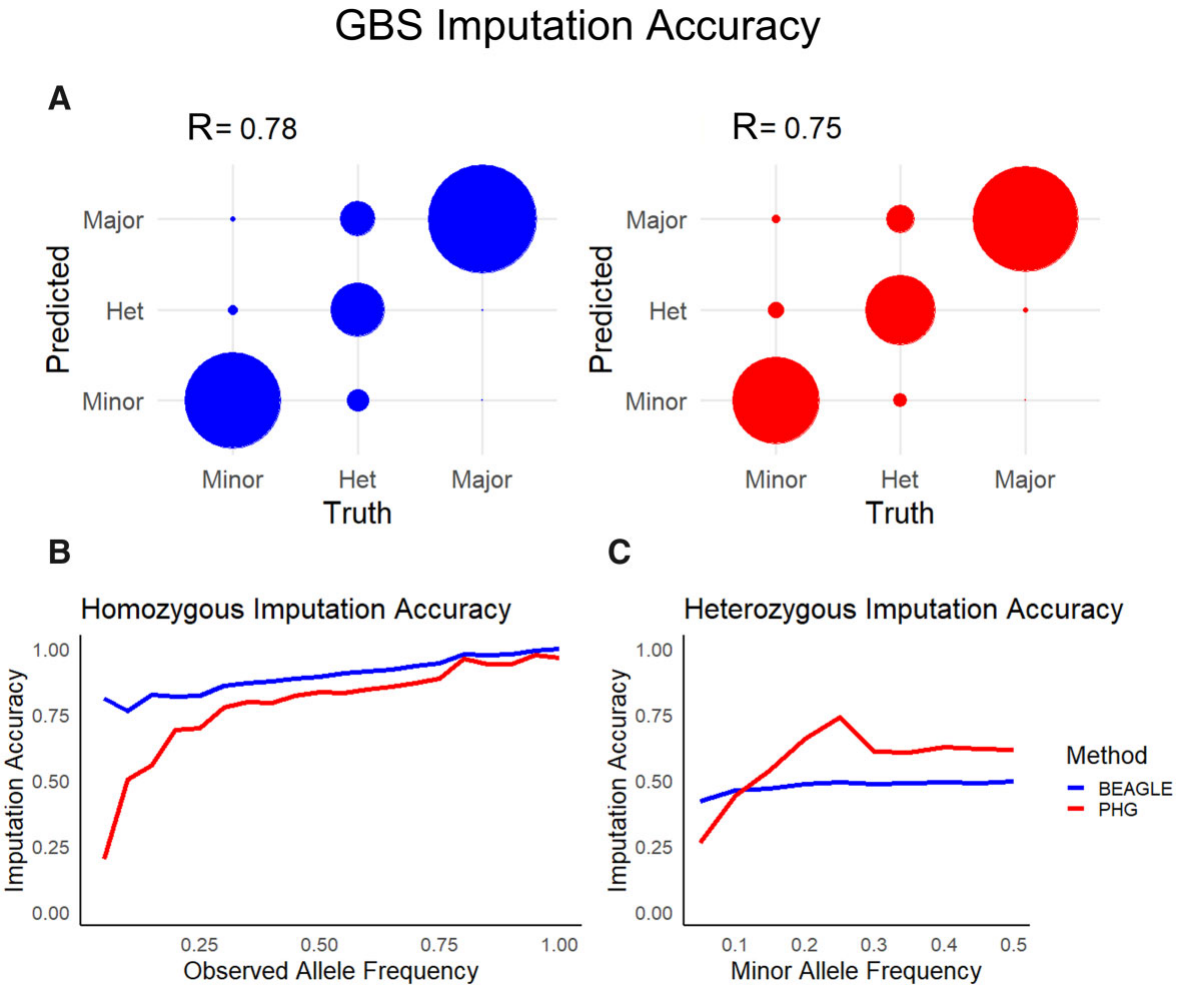
Imputation accuracy from GBS sequencing. (A) Displays concordance between true and imputed alleles separated by alleles classes (circle radius is equal to the proportion of alleles in each class). (B) Imputation accuracy is shown for homozygous genotypes separated by allele frequency of the true allele at that locus. (C) Imputation accuracy is shown for heterozygous genotypes separated by minor allele frequency at that locus.

In addition to skim sequence scenarios, we also tested imputation using available GBS sequence for 20 clones. While skim sequence samples a random set of reads from across the genome, GBS is a replicable set of markers that a sparsely sampled across the genome. Imputation tests showed similar, but somewhat reduced accuracies using the PHG compared to Beagle (Figure  5A). It is important to note, however, that the PHG still had improved accuracies in imputing heterozygous genotypes (Figure  5C).

**Figure 6:**
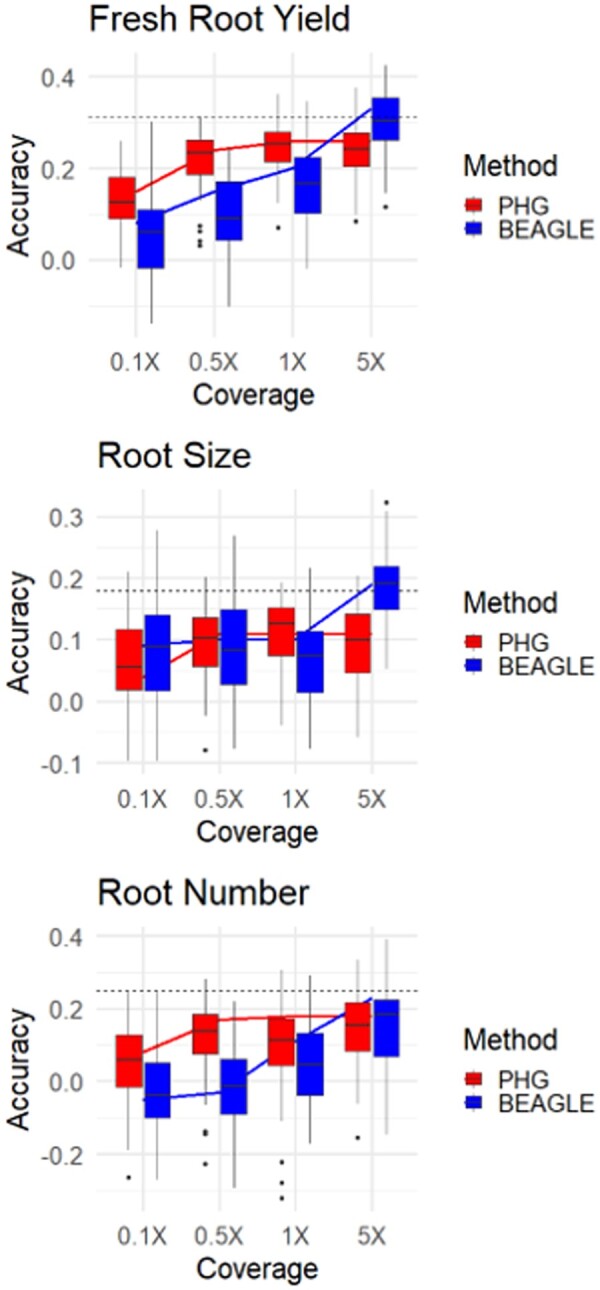
Genomic prediction cross-validation. Tenfold cross-validation (box) and single holdout cross-validation (line) show genomic prediction accuracies of three root traits using different imputation methods at varied sequence depths. Single holdout cross-validation using complete genotype dataset is shown (dashed line).

The imputed genotypes from skim sequence were then utilized in a genomic prediction scheme consisting of 57 cassava clones (Supplementary Figure S4) from a single breeding program. Clones were selected from a single breeding program to minimize confounding factors such as population structure and ensured an adequate level of heritability to assess genomic prediction accuracy. Tenfold cross-validations and leave-one-out validation showed that imputation accuracy generally appeared to follow the trends in genomic prediction accuracy, for fresh root yield and root number, while no clear pattern was apparent for the root size trait (Figure  6).

### Phased haplotype PHG

**Figure 7:**
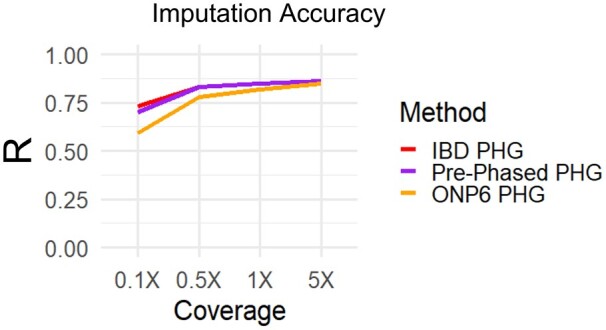
Haplotype phasing methods in the PHG. Imputation accuracy is shown for three different methods of populating a PHG. First, the IBD PHG (red) was populated using homozygous haplotypes from the 241 HapMapII clones. Second, the Prephased PHG (Purple) used Beagle and HPACUT2 to phase these same clones. Third, the ONP6 PHG (Yellow) used ONP long reads and WhatsHap to phase six related taxa to the test set.

We tested the viability of populating the PHG with haplotypes phased by other methods. We compared the IBD method of sampling phased haplotypes to two methods of phasing variants. The first method used Beagle and HAPCUT2 to phase the variants called from the HapMapII WGS data. The second method utilized six cassava clones with ONP long-read data. The IBD and prephased methods of populating the cassava PHG produced almost identical accuracies (Figure  7). These results suggest that Beagle and HAPCUT could not accurately phase heterozygous haplotypes at this scale, and the accurate haplotypes are derived from IBD haplotypes. While the PHG was made from six clones with ONP data did perform as well as the other methods, it relied on a far narrower set of germplasm. This suggests that accurate haplotypes were likely captured using this method but lacked adequate sampling to capture sufficient haplotypes.

### Imputation simulation

**Figure 8:**
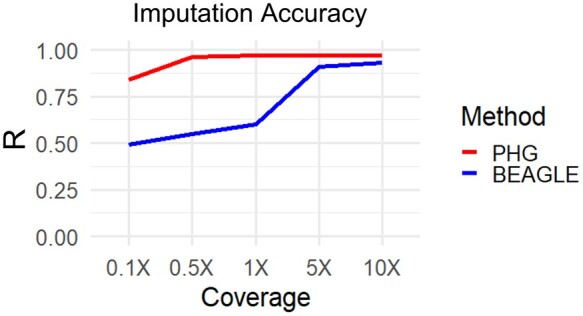
Imputation accuracy with simulated genotypes. A simulated scenario where 20 parents with full phased information are used to populate a PHG. Correlation between imputed and true variants by imputing with the PHG and Beagle at different levels of skim sequencing.

Evident from the tests using haplotypes from IBD regions of the genome, sampling phased haplotypes is a difficult aspect of creating an effective PHG in a heterozygous species. To explore the performance of the PHG in a scenario where one could aptly sample the diversity of haplotypes, we used simulated offspring from a set of 20 phased genomes. While phasing errors exist, we accepted these phases as truth for the simulation of offspring. This ensured that all haplotypes present in the offspring exist in the PHG database. We found that the disparity in accuracies between PHG and Beagle at high sequence coverage disappeared in our simulation (Figure  8), while the trend in Beagle accuracy was very similar to our empirical tests. While the simulation does represent an ideal scenario, including a narrower set of germplasm, it highlights the performance of the PHG when accurately phased haplotypes are available.

## Discussion

We have detailed a method of implementing a PHG for the heterozygous plant species cassava. This PHG database utilizes phased haplotypes to predict missing genotypes from low-depth input sequence. Runs of homozygosity formed by IBD relationships proved to be a reliable method of sampling phased haplotypes given the available data (Figure  7). This method of obtaining haplotypes, while not able obtain the full diversity of alleles, captured 77% of common alleles and produced ample haplotypes for significant imputation accuracy at very low sequence depth (Figure  4A).

The high accuracy of the PHG demonstrates its potential as an imputation tool for use in heterozygous crops. The advantages of the PHG imputation methodology are especially evident in its accuracy at calling rare and heterozygous alleles (Figure  4, C and D). Furthermore, the observed weaker relationship between allele frequency and imputation accuracy, highlights its ability to predict rare alleles. Across both simulated and empirical experiments, we found that the ability of the PHG to impute whole-genome variants was consistent at or above 0.5× sequence coverage. The haplotype-based representation of the genome enables this imputation methodology to overcome the logistical hurdles such as those produced by sequencing and assembly errors, repetitive sequences, and poor alignments.

The plateau reached in imputation accuracy (Figure  4A) using the PHG most likely indicates that we have not sufficiently sampled the diversity of possible haplotypes. At sequence coverages of 5× and higher, the raw data can produce the true genotypes and little imputation of missing genotypes is occurring. The PHG imputation is limited to predicting haplotypes that are already present in the database, while Beagle can rely on the genotypes called from the high depth (>1×) raw sequence, meaning that there are much fewer missing data for Beagle to impute. This scenario of high depth sequence is useful to diagnose challenges in imputation, however it does not correlate to many real applications. The disparity between the PHG and Beagle at these high coverages points to the presence of missing haplotypes in the database, rather than any disparity in performance.

The hypothesis of missing haplotypes limiting imputation accuracy is supported by a visible relationship between homozygous incidence in our population and reference range imputation accuracy (Supplementary Figure S5), suggesting that those ranges with poor imputation accuracy were not amply sampled. The length and abundance of the IBD runs of homozygosity in our dataset likely determine the ability of the HMM to accurately predict haplotypes. There may be many factors that affect the prevalence of IBD haplotypes including recessive deleterious effects, populations size, population diversity, and heterozygosity. We saw that the disparity in imputation accuracy was remedied under simulation, where all possible haplotypes were sampled in the database (Figure  8). These results suggest that, although an already powerful tool, the PHG achieves maximum performance with sufficient sampling of available haplotypes.

Currently, the performance using GBS data appears to be similar between the PHG and Beagle (Figure  5). Imputation from reduced representation genotyping such as GBS is challenging due to the sparse sampling across the genome and varied levels of sequence quality. Excellent imputation accuracy in inbred crops Sorghum (Jensen *et al.* 2020) and Maize (Valdes Franco 2020) using these genotyping methods highlights the potential benefits of the PHG in these scenarios. Because reduced representation genotyping methods are likely the most commonly implemented, current efforts are being made to improve heterozygous imputation using these technologies. We expect improved haplotype sampling and phasing to improve imputation accuracy for these genotyping platforms. Further haplotype sampling paired with developments in the PHG imputation methodology will likely improve imputation accuracy from these genotyping methods.

While the imputation accuracy of the PHG is limited based on the haplotype sampling, its high accuracy with low levels of input sequence highlights its potential for genomic applications, where sparse genotyping is common. We showed that this is true regarding genomic prediction by performing cross-validations with the imputed genotypes (Figure  3). The genomic prediction was still limited by imputation accuracy, but by enabling higher accuracy we can achieve more reliable predictions (Pimentel *et al.* 2015; Wang *et al.* 2016; Van Den Berg *et al.* 2017).

With increased imputation accuracy from more limited genotyping inputs, a breeding program may be able to afford to cross and genotype more offspring, enabling them to increase selection pressure across their breeding pool. Similarly, imputation to genome-wide scale can bridge gaps between different data sets containing information on different marker panels, enabling the use of larger datasets for prediction. Accurate imputation could also enable breeders to utilize genomic prediction models that incorporate more prior functional information on genome-wide variant effects into predictions, using methods such as GFBLUP (Fang *et al.* 2017) or BayesR (MacLeod *et al.* 2016; Van Den Berg *et al.* 2017). These possible applications of imputation have the potential to increase total genetic gain made by breeding programs.

We show that while computational methods might not be able to solve haplotype phasing with short-read data, long-read sequencing may be able to overcome that issue. The Prephased PHG produced similar accuracies to the IBD method, suggesting the additional haplotypes added by phasing why heterozygous alleles using Beagle and HAPCUT were not accurate over long distances. While limited in scope, the ability of the PHG created from six clones with ONP data suggests the potential application of long reads for obtaining phased haplotypes. One could envision a breeding scenario in which parents are sequenced and phased using long-reads and offspring are predicted from minimal genotyping input using the PHG. Then every few generations shallow WGS can be used to update the PHG and compensate for changing LD structures.

Applying the PHG to cassava and other heterozygous crops will depend on the ability to sample phased haplotypes within the given population. We’ve shown that utilizing high depth WGS data and IBD regions of the genome can be used to reliably sample many phased haplotypes, and that the resulting PHG can impute with high accuracy from low-depth sequence. This method of sampling haplotypes will be highly dependent on the diversity and heterozygosity of the species and population for any given application. Other necessary considerations for the decision to use the PHG include genome size, reference genome quality, training data availability, species ploidy. In applications where imputation is commonly implemented, training data that can be used to construct a PHG may already be available. Our long-read data results show the potential for more easily capturing phased haplotypes as these technologies become more available. Using genome assemblies produced from long-reads as inputs to the PHG has been shown to be very effective in Maize, while this method has not been implemented in outbred species. The potential for the PHG as a tool in heterozygous crops has been shown here, while the specific methods to produce the phased haplotypes will have to be designed around the target species and scenario.

## Conclusion

The PHG is a method to reduce a genome to a set of haplotypes. We have shown that this method can be used to predict whole-genome haplotypes in a heterozygous species from sparse genotyping information. Its high accuracy, especially in rare alleles, at very low depths of skim sequencing makes it a potentially powerful imputation tool. Continued work in populating the PHG database with confidently phased haplotypes will lead to a more consistent prediction model across varied genotyping methods.

## Data availability

Supplementary files and scripts used for the production and testing of the cassava PHG can be found at https://bitbucket.org/bucklerlab/p_cassava_phg. Genotype and phenotype data from HapMapII (Ramu *et al.* 2017) were downloaded from cassavabase.org. Support and methods for PHG implementation can also be found at https://bitbucket.org/bucklerlab/practicalhaplotypegraph/wiki/Home. Raw ONP sequence data for this project are available at NCBI BioProject ID PRJNA589272.

Supplementary material is available at *G3* online

## Supplementary Material

jkab383_Supplementary_Figures

jkab383_Supplementary_Figures-Captions
