# Supplementary figures and images for: Genome-wide imputation using the practical haplotype graph in the heterozygous crop cassava
Source: G3 (Bethesda). 2021 Nov 9;12(1):jkab383. doi: 10.1093/g3journal/jkab383 (PMC8728015; doi:10.1093/g3journal/jkab383)

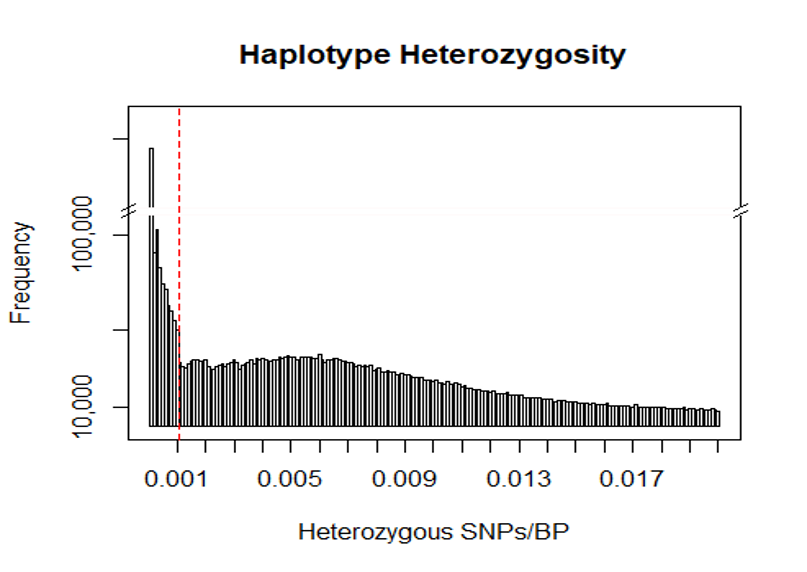

Supplement: jkab383_Supplementary_Figures [file jkab383_supplementary_figures.zip › GENETICS-G3-2021-402876-s01.png]

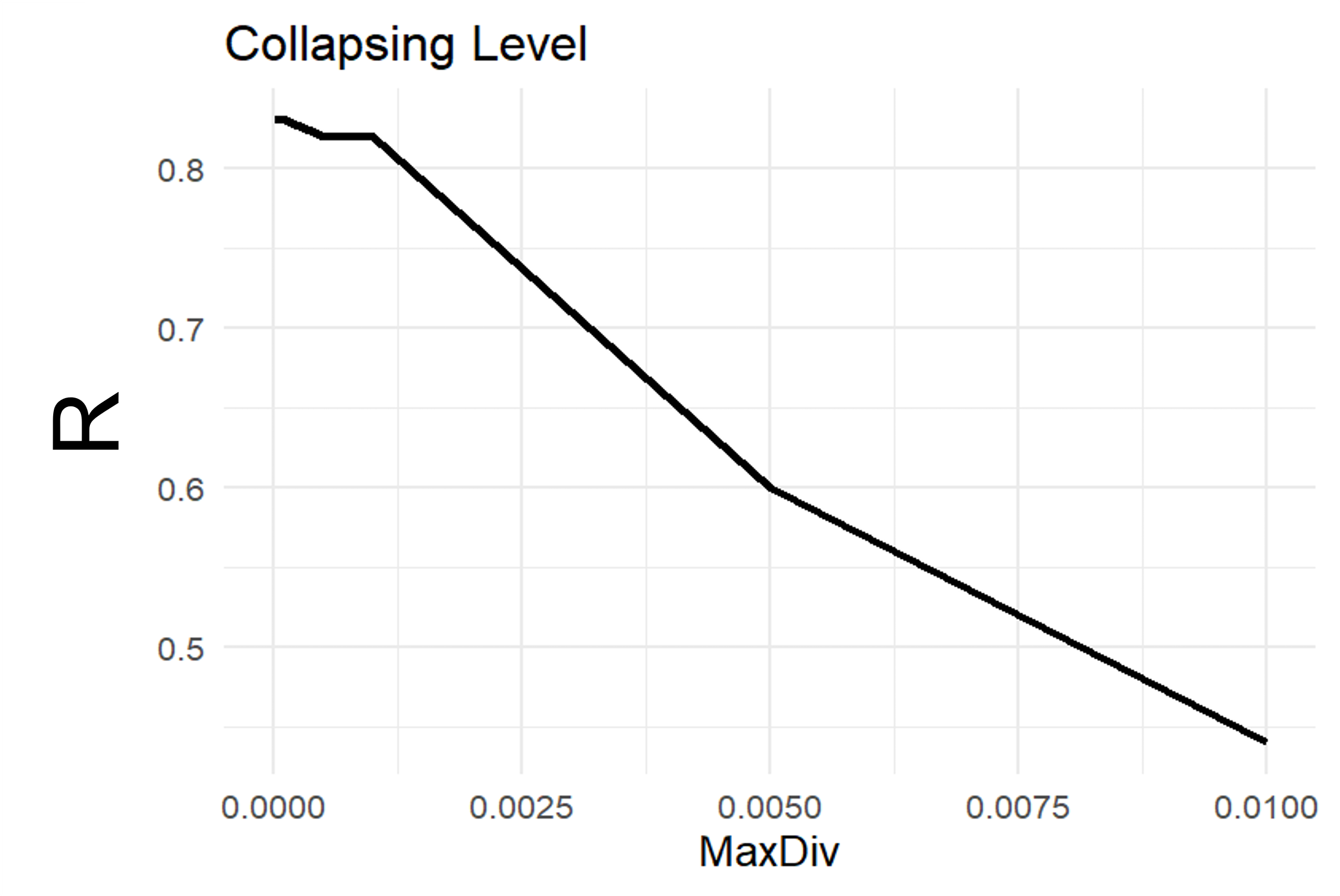

Supplement: jkab383_Supplementary_Figures [file jkab383_supplementary_figures.zip › GENETICS-G3-2021-402876-s02.png]

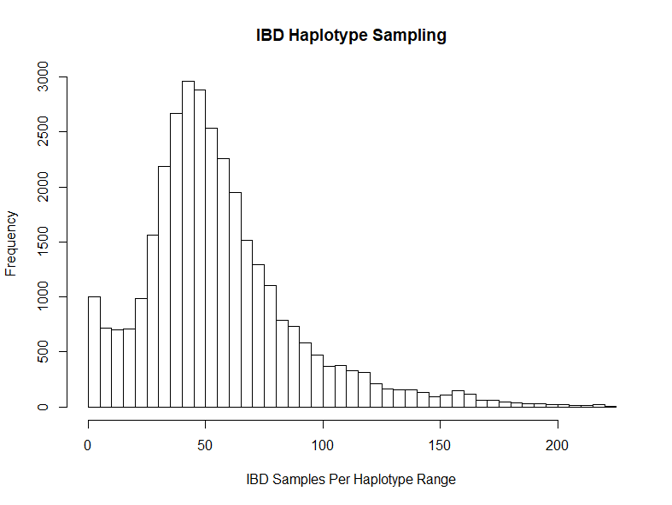

Supplement: jkab383_Supplementary_Figures [file jkab383_supplementary_figures.zip › GENETICS-G3-2021-402876-s03.png]

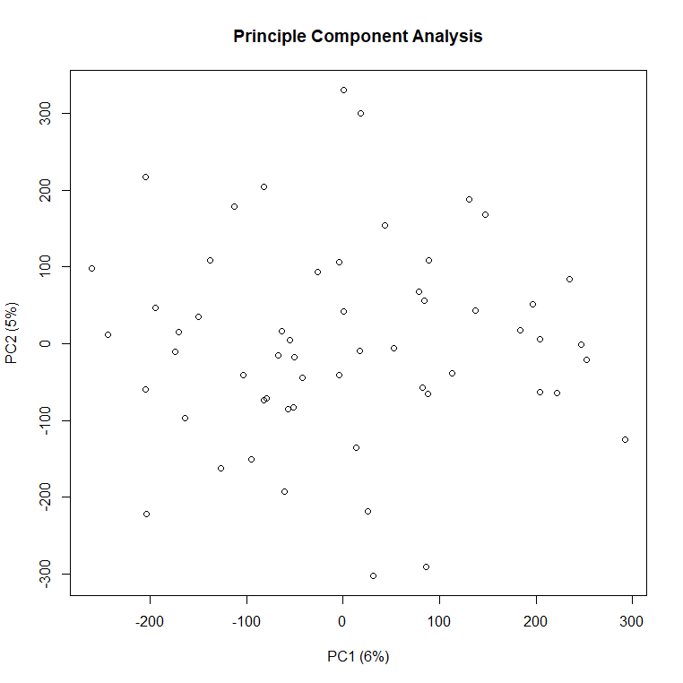

Supplement: jkab383_Supplementary_Figures [file jkab383_supplementary_figures.zip › GENETICS-G3-2021-402876-s04.png]

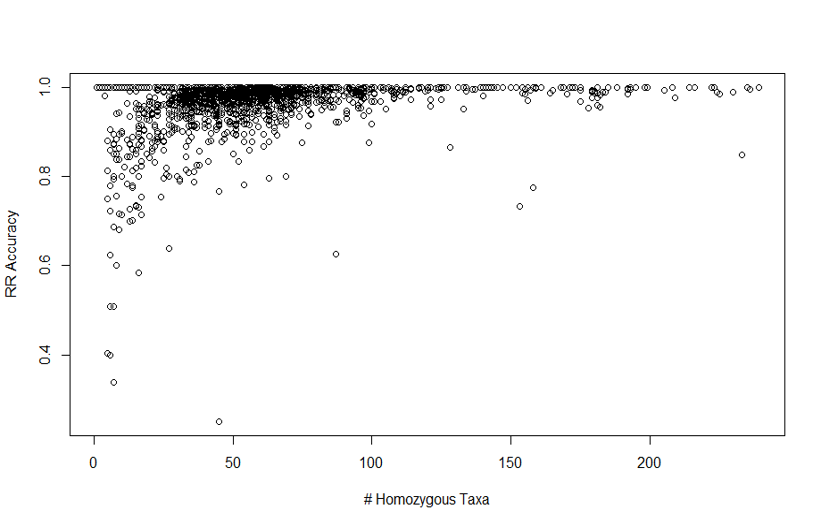

Supplement: jkab383_Supplementary_Figures [file jkab383_supplementary_figures.zip › GENETICS-G3-2021-402876-s05.png]
