# Supplementary material for: Genome-wide imputation using the practical haplotype graph in the heterozygous crop cassava
Source: G3 (Bethesda). 2021 Nov 9;12(1):jkab383. doi: 10.1093/g3journal/jkab383 (PMC8728015; doi:10.1093/g3journal/jkab383)
Supplement: jkab383_Supplementary_Figures-Captions [file jkab383_supplementary_figures-captions.pdf]

SUPPLEMENTAL FIGURES

Supplemental Figure 1. Histogram of heterozygous SNPs per base pair is shown at haplotypes at each reference range from each taxon. Dotted line shows the threshold chosen to distinguish homozygous haplotypes.

Supplemental Figure 2. Correlations of Imputed calls to true calls at different haplotype collapsing levels based on the maximum divergence parameter of the PHG.

Supplemental Figure 3. Histogram of IBD sampling frequency of all reference ranges. Y-axis shows the number of reference ranges with a given number of IBD samples.

Supplemental Figure 4. Principal component analysis of 57 clones used in genomic prediction cross validation. Lack of clusters show little population structure among the clones.

Supplemental Figure 5. Imputation accuracy at each reference (y-axis) range by homozygous incidence in the HapMapII Population (x-axis). Low accuracy shown at reference ranges with low incidence of homozygous taxa.
